# Progress in Clinical Trials of Photodynamic Therapy for Solid Tumors and the Role of Nanomedicine

**DOI:** 10.3390/cancers12102793

**Published:** 2020-09-29

**Authors:** Hashem O. Alsaab, Maha S. Alghamdi, Albatool S. Alotaibi, Rami Alzhrani, Fatimah Alwuthaynani, Yusuf S. Althobaiti, Atiah H. Almalki, Samaresh Sau, Arun K. Iyer

**Affiliations:** 1 Department of Pharmaceutics and Pharmaceutical Technology, College of Pharmacy, Taif University, Taif 21944, Saudi Arabia; r.zhrani@tu.edu.sa; 2 Department of Pharmaceutical Care, King Abdul-Aziz Specialist Hospital (KAASH), Taif 26521, Saudi Arabia; MAlghamdi372@moh.gov.sa; 3 College of Pharmacy, Taif University, Al Haweiah, Taif 21944, Saudi Arabia; albatool.saad@hotmail.com (A.S.A.); Falwuthaynani@moh.gov.sa (F.A.); 4 Department of Pharmacology and Toxicology, College of Pharmacy, Taif University, Taif 21944, Saudi Arabia; ys.althobaiti@tu.edu.sa; 5 Department of Pharmaceutical chemistry, College of Pharmacy, Taif University, Taif 21944, Saudi Arabia; ahalmalki@tu.edu.sa; 6 Use-Inspired Biomaterials and Integrated Nano Delivery Systems Laboratory, Department of Pharmaceutical Sciences, Eugene Applebaum College of Pharmacy and Health Sciences, Wayne State University, Detroit, MI 48021, USA; gi7517@wayne.edu (S.S.); arun.iyer@wayne.edu (A.K.I.); 7 Molecular Therapeutics Program, Barbara Ann Karmanos Cancer Institute, School of Medicine, Wayne State University, Detroit, MI 48201, USA

**Keywords:** photodynamic therapy/PDT, cancer, solid tumors, nanotechnology, photoimmunotherapy, photosensitizing agents

## Abstract

**Simple Summary:**

Photodynamic therapy (PDT) has been undertaken with growing focus in recent studies to identify successful anticancer therapies. The field of PDT has evolved rapidly and is continuously being evaluated with new techniques. To make PDT more active and selective, molecular strategies are being developed. In the latest clinical studies on the use of PDT, some challenges are presented. Therefore, the use of nanotechnology-based approaches as delivery tools for PSs may improve their cancer cellular uptake and their toxic properties, as well as the PDT’s therapeutic impact. In addition, photoimmunotherapy (PIT) and photothermal therapy (PTT) might have a significant impact on solid tumor therapeutic strategies.

**Abstract:**

Current research to find effective anticancer treatments is being performed on photodynamic therapy (PDT) with increasing attention. PDT is a very promising therapeutic way to combine a photosensitive drug with visible light to manage different intense malignancies. PDT has several benefits, including better safety and lower toxicity in the treatment of malignant tumors over traditional cancer therapy. This reasonably simple approach utilizes three integral elements: a photosensitizer (PS), a source of light, and oxygen. Upon light irradiation of a particular wavelength, the PS generates reactive oxygen species (ROS), beginning a cascade of cellular death transformations. The positive therapeutic impact of PDT may be limited because several factors of this therapy include low solubilities of PSs, restricting their effective administration, blood circulation, and poor tumor specificity. Therefore, utilizing nanocarrier systems that modulate PS pharmacokinetics (PK) and pharmacodynamics (PD) is a promising approach to bypassing these challenges. In the present paper, we review the latest clinical studies and preclinical in vivo studies on the use of PDT and progress made in the use of nanotherapeutics as delivery tools for PSs to improve their cancer cellular uptake and their toxic properties and, therefore, the therapeutic impact of PDT. We also discuss the effects that photoimmunotherapy (PIT) might have on solid tumor therapeutic strategies.

## 1. Introduction

**Figure 1:**
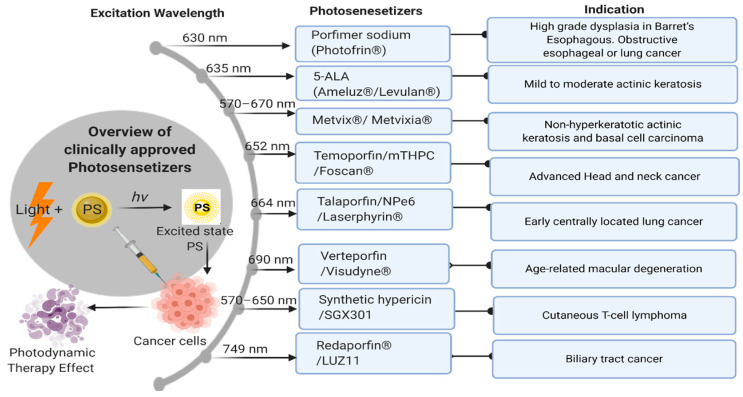
The mechanism of photodynamic therapy (PDT) effects and a list of clinically approved Photosensitizers (PSs) with their excitation wavelengths and indications.

The global rise in the incidence of cancer has led to an increase in the need for safe and effective treatment materials. Photodynamic therapy (PDT) is considered an alternative to radiation therapy and chemotherapy, which are the most common forms of cancer treatment. Researchers have been exploring new strategies based on nanotechnology to increase PDT efficiency in recent years [1]. PDT is a procedure that has been proven to be extremely useful for the treatment of many forms of cancer and is considered to be a minimally invasive approach [2]. PDT injects photosensitizers (PSs) into the blood supply or the tumor directly through light-sensitive treatment as shown in Figure 1. Such substances include chromophore molecules that transfer their energy as they are irradiated into cells with oxygen and contribute to the development of singlet or other reactive oxygen species (ROS), which may inflict substantial harm to the cells or blood tumors and stimulate the immune system’s anticancer activity [3]. 

As PDT has been developing since 1980, many other therapeutic options have improved. PDT has been proven to be useful in many types of tumors [4], such as esophageal cancer [5], melanoma [6], and multidrug-resistant breast cancer [7]. PDT also has antibacterial properties [8] and can be widely used in actinic keratosis, minor skin disease [9], or Condyloma acuminatum [10]. For PDT to be considered the best option, it will need to be equal to or greater than other treatments or provide an additional impact when used in combination with new therapies [11]. However, PDT is not without drawbacks, particularly its role in impaired cellular uptake and the weak biodistribution of PSs. Indeed, the off-target accumulation of PSs may cause mild but long-lasting phototoxicity, such as light sensitivity leading to burns, swelling pain, and scarring in the normal tissues near the tumor area. The other barrier is poor light penetration through the tissues, limiting the use of PDT for skin cancer, and the superficial small tumors found on or just below the surface [11].

The goal of current research is to expand PDT to various kinds of cancer and to produce more efficient PSs and targeted delivery methods. The use of nanomaterial delivery technologies that can modify biodistribution along with the PK and PD characteristics of PSs is among the most promising techniques [12]. In this regard, the quickly evolving field of nanomedicine and nanotechnology can produce nanostructured materials lacking the drawbacks of delivery systems that are used clinically. Nanoparticles are capable of protecting medications from degradation along with increasing their solubility, prolonging their blood half-life, and facilitating targeted delivery and cellular uptake. This makes nanostructures an exciting alternative to traditional PDT, as they enable the transport and penetration of PSs and can ameliorate extreme anticancer activity [12]. To date, numerous different nanoparticles have been studied for PS delivery to increase their concentration in the cancer area, as well as their phototoxic properties, such as liposomes, dendrimers, gold nanoparticles, silver nanoparticles, and polymersomes [13]. Some of these nanoparticles are currently being translated through in vitro to in vivo experiments with the ultimate goal of developing new PDTs in polymers, metals, and silica carriers. Such materials can be used to promote the development of tumors and boost the phototoxic effects of drugs in animal models [14].

In the present study, we reviewed the latest clinical studies and in vivo studies on the use of PDT and the progress of the use of nanoparticles as potential delivery tools for PS delivery to improve their cellular uptake and cytotoxic properties and, therefore, the therapeutic impact of PDT.

## 2. Photodynamic Therapy Mechanism and Advantages

There are currently numerous cancer treatments available, including chemotherapy, radiation, surgery, monoclonal antibodies, immunotherapy, and different combinations [15]. The main problem in using most conventional therapies for cancer, such as chemotherapeutics and radiation, is their low specificity for cancer cells combined with their often high toxicity to non-specific cells of the cancer patients [16,17]. The choice of treatment is primarily determined by the type and phase of the disease and by the overall health condition of the patient [18,19]. PDT is a promising alternative method for treating a variety of cancer diseases involving the destruction of abnormal cells, which requires the application of PS to specific dysfunctional cells and the placement of light at an appropriate wavelength to activate PS [19]. PS photodynamic activity is based on photooxidant reactions that cause many subsequent biochemical and molecular reactions [20]. This allows cytotoxic reactive oxygen (ROS), such as single molecular oxygen, to be released [21,22]. Studies indicate that there is no particular mechanism leading to cell death after PDT. PDT causes cancer oxidative damage via three main pathways of cell damage: apoptosis, necrosis, and autophagy. These pathways can occur alone or concurrently [11,20]. PDT may also function by disrupting tumor-related vasculature, tumor infarctions, and reprogramming the immune system to attack the cancer cells [23].

Photodynamic activity depends on the quality of the PS used, its localization (both extracellular and intracellular), the total dose given, the overall light intensity, the time interval between drug administration and exposure to light, the oxygenation state of the tissue, and the cell types affected [24]. PDT could be an early or localized curative modality for cancer, thereby improving quality of life and prolonging the survival of those with advanced stages of the disease. PDT’s benefits usually involve cost-effectiveness, highly localized therapies, extracellular matrix sparing, and the duration of the treatment without toxicity. Further, combinations of PDT with chemotherapy and immunotherapy might lead to stronger treatment responses and immune enhancement, which may contribute to long-lasting tumor size management and treatments that can be given to outpatients [25,26]. There are certainly other benefits of PDT over the various types of chemotherapy against cancer. Due to the use of PS and a specific light source, reactions can only be stimulated at a certain point and time. The wide range of available phototoxic compounds allows one to choose the right treatment strategy [27,28]. Nevertheless, in most cases, the positive therapeutic outcome is constrained by several factors, despite a large number of studies and great efforts being made in developing and optimizing successful PDT, such as anticancer modality [3]. PDT’s drawbacks include impaired cellular uptake and the weak biodistribution of highly lipophilic PSs. Indeed, the off-target accumulation of PSs may yield mild but long-lasting problems such as skin photosensitivity leading to burns, swelling pain, and scarring in the normal tissues near the tumor environment [29]. Therefore, after PS administration, reticuloendothelial (RES) cells can rapidly degrade or eliminate PSs from the tumor microenvironment [3]. Another barrier is poor light penetration through tumor tissues, making the applicability of PDT to very deep solid tumors very difficult [11]. Moreover, PDT’s anticancer activity may also be reduced due to low tumor selectivity and activation energies requiring prolonged illumination times.

## 3. Clinical Application of PDT for Solid Tumors

PDT’s therapeutic use in cancer began in the late 1970s, when five patients with bladder cancer were tested to determine the effects of Hematoporphyrin derivative (HPD) + light [30,31]. In 1978, Dougherty reported the first large successful cohort of PDT patients with HPD [3]. In 111 out of 113 malignant tumors, full or partial effects were observed. None were found to be unresponsive among the wide range of tumors examined. In 1978, in a woman with metastatic breast cancer of the skin, Dougherty et al. reported the first clinical application of PDT, and H. Kato et al. began a study of PDT using a hematoporphyrin derivative and a krypton ion laser or argon dye laser [32,33]. More than 250 PDT clinical trials have been conducted since this initial work. Recent systematic reviews [34,35] have shown that PDT may be regarded as an appropriate treatment option for malignant and pre-malignant skin cancers. Barrett’s esophagus and unresectable cholangiocarcinoma are also useful to be treated by PDT. However, PDT has not yet been unequivocally demonstrated to be effective in the treatment of other types of tumors. The main reason for this lack of information is that only a few randomized controlled trials with adequate power have been conducted.

Systematic literature analysis is limited due to the absence of optimum PDT parameters among the studies (illumination states or PS doses). PDT primarily causes superficial effects. The depth of tumor destruction ranges from just a few millimeters to one centimeter due to limited light penetration through tissues. This obvious drawback can be used in treating surface diseases such as premalignancies (mucous, actinic keratosis), on-site carcinoma, and surface tumors (for instance, malignant pleural mesothelioma [36] or intraperitoneal disseminated carcinomatosis [37,38]). PDT may also be used as an addition to surgery to irradiate tumor beds and increase the possibility of local disease control in the long term. Furthermore, PDT for several upper GI tumors has shown positive results in some clinical trials [39]. Although many clinical trials have been conducted on the use of PDT in malignant brain tumors [40], most of these were phase I/II trials. The heterogeneity of the procedures, adjuvant therapies, and subtypes of tumors used in these studies further hinders the estimation of PDT’s effectiveness. PDT may also be used for tumor irradiation and the probability of local disease control over long periods [31]. The PDT procedure is used to treat many types of solid tumors. As shown below, we discuss some but not all of the solid tumors that might benefit from utilizing a PDT modality of treatment. 

### 3.1. Brain Tumor

The most significant pathological characteristic of glioblastoma, typical of malignant brain tumors, is its infiltrative nature. Since normal brain tissues and tumor cells coexist at the tumor–brain interface, complete tumor cell resection requires the loss of healthy brain tissue. Moreover, because isolated brain functions are not replaceable at any other location, resections of the tumor must be relinquished if a tumor infiltrates the functional areas of language, motor functions, senses, vision and memory. The tumor will then return, grow, and kill the patient [41,42]. In comparison to surgery and radiation, PDT can target micro-invasive areas and also spare susceptible regions of the brain [43]. This advantage over existing treatments can improve results for patient populations with a generally poor survival and incidence of iatrogenic injury. In addition, the inducible nitric oxide synthase (iNOS) inhibitors, the administration of specific types of EGFR inhibitors, and nanoparticles show promise for improving the effectiveness of PDT in the treatment of brain cancer [43]. In an interventional study of Photofrin (porfimer sodium) and PDT, five patients with relapsed or refractory brain tumors, even if the tumors were supratentorial or infratentorial (posterior fossa) in their location, received Photofrin through an IV infusion around 24 h before their tumor resection surgery and PDT. PDT involved photo illumination at 630 nm starting at the center of the tumor resection cavity and obtaining a total energy of 240 J·cm^−2^. After tumor removal, the optical fiber was placed in the approximate center of the surgical cavity. Then, the intralipid was administered into the open tumor cavity while PDT was conducted. The Intralipid diffused the light and ensured uniform delivery. For brain tumor patients, the methodology conducted in this study used higher intensity laser light and higher PS doses of Photofrin than previous PDT protocols in the United States. The team conducting the trials suggested that the light should deeply penetrate far enough into the solid tumors to reach migrating cancer cells and kill them without affecting the normal cells. The authors tested the assumption that pediatric participants with progressive/recurrent malignant brain tumors undertaking PDT with increasing doses of Photofrin^®^ and light energy would have better progressive free survival (PFS) and overall survival (OS) results than those in the previous clinical study. The specific objectives included evaluating the maximum tolerable dose (MTD) of Photofrin in pediatrics and looking for preliminary response trends (NCT01682746).

The same idea was discussed in a study done at the Royal Melbourne Hospital, which was published in 2005. The investigators measured the survival time as a study outcome after exposing high-grade glioma patients to PDT. A total of 136 patients had a tumor resection for glioblastoma/GBM (78 patients) and anaplastic astrocytoma/AA (58 patients) at the hospital between 1986 and 2000 and received 5 mg/kg of a haemetaporphyrin derivative (HPD) as a photosensitizer. Then, the patients were exposed to laser light [44]. The survival time was longer than 36 months for 73% and 25% of the newly diagnosed patients with AA and GBM, respectively. Patients with recurrent AA (57%) and GBM (41%) after repeat surgery also survived beyond 36 months. This study defined old age at the time of diagnosis as one of the factors responsible for a poor prognosis. Skin photosensitization and cerebral edema were the most probable complications of PDT [44]. 

In 2019, Mahmoudi and his team published a comprehensive review that summarized five clinical trials that aimed to study the efficacy and safety of using 5-Aminolevulinic acid (5-ALA) with PDT in patients with high-grade gliomas (HGGs) [45]. A longer survival time was reported by the end of these studies [46,47,48,49,50], which were uncontrolled phase I and II studies with few participants. That was why the authors could not generalize the results or confirm the treatment’s safety. For that, Mahmoudi et al. recommended conducting more large, multicenter, randomized controlled Phase III trials to clarify the role of 5-ALA photosensitizers and PDT and confirm their importance as strategies for glioma treatment [45]. Many clinical trial examples are noted in Table 1.

### 3.2. Lung Cancer

PDT is a well-established method for treating non-small cell lung cancers (NSCLCs). Newly introduced PSs, which have shown promising outcomes in phase I and II clinical trials, are expected to minimize phototoxicity, which is the main adverse event [51,52]. In 1980, the first endoscopic PDT procedure in lung cancer patients with poor cardiopulmonary function who could not undergo surgery was performed. In March 1980, the second case involved early-stage squamous cell carcinoma of the upper right bronchus [53,54]. The patient was a man aged 74 who declined surgery, making this the world’s first case of PDT for early-stage lung tumors. There was a complete cure, and the man remained disease-free for >5 years [55].

In one of the PDT studies for lung cancer (NCT00984243), which was an interventional open-label study and included 35 participants with squamous cell lung cancer, Photofrin II was administered with a dose of 2 mm/Kg i.v. Laser therapy with an argon-dye or excimer-dye laser was carried out 40–50 h later at 620–630 nm. This study aimed at determining whether PDT is a replacement for surgical resection in cancer patients who are candidates for the procedure with early-stage squamous cell lung carcinoma. If PDT were successful, it would remove the need for surgery and operation. The primary objectives were to assess the effects of PDT on lung cancer patients by measuring the percentage of patients spared surgery, as well as their morbidity, overall mortality, risk of recurrent lung cancer, improvements in pulmonary function over time, impact on quality of life, and preferences.

Another group studied PDT during surgery for treating patients with NSCLC that can be removed by surgery (NCT01854684). In this phase I trial, eight patients with resectable primary NSCLC who underwent surgery to resect their T3 to T4 lesions and patients with clinical NI or N2 disease independent of the T-stage were provided temoporfin intravenously (IV) for at least 6 minutes and then underwent normal intraoperative PDT. After completion of the study treatment, patients had to follow up every six months for two years. The primary objective was to demonstrate the use of intraoperative adjuvant regional PDT with a low dose. The secondary objectives of this study were an initial assessment of efficacy (i.e., two-year disease-free survival), to measure the light dose and the clinical outcome, and to measure temoporfin uptake in malignant and normal tissue. Many clinical trial examples are outlined in Table 1.

### 3.3. Urological Tumors: Bladder Cancer 

PDT is used for the treatment of diffuse superficial transitional-cell bladder carcinoma refractory to standard therapies through interactions between absorbed light and a retained light-sensitive agent to destroy cancerous tissue. One downside is that light stimulates the chemicals such that cancer cells can only be affected close to the surface of the bladder lining. Moreover, the light cannot penetrate deeper bladder tumors. However, there are few reports of long-term outcomes [56]. PDT has been used as a successful treatment for in situ refractory carcinoma and recurrent transitional papillary cell carcinoma. Clinical trials have been conducted to evaluate whether this form of therapy is better than standard BCG immunotherapy or chemotherapy [57]. While PDT was useful for bladder cancer, due to the non-specific treatment of urothelium, its application was complicated by toxicities. In this regard, bladder cancer photoimmunotherapy is a more targeted form of PDT that allows selective tumor cell destruction with less toxicity [58].

Several clinical trial findings for bladder PDT have been published, as mentioned in Table 1. In an interventional study (NCT03053635) of TLD1433 infusion and PDT treatment, subjects with BCG refractory high-risk non-muscle-invasive bladder cancer (NMIBC) who were not suitable for, or refused, radical cystectomy were used for conducting this study, which was started in 2017. Six patients received TLD1433, which was infused into the bladder along with treatment of the bladder wall with PDT. The key results of this study were a TLD1433 safety analysis and assessment of PDT’s adverse effects, incidence, and severity, as well as a secondary outcome measure of pharmacokinetics. Another exploratory outcome endpoint was also measured, recurrence-free survival (RFS).

Another group in an interventional phase I trial (NCT01303991), featuring 17 patients with intermediate or high-risk bladder cancer, was treated with Hexvix PDT using Karl Storz T-Light. The study aimed to determine the safety and feasibility of Hexaminolevulinate-based PDT in transitional cell bladder carcinoma patients with intermediate or high-risk disease. Another group in an interventional study phase I and phase II trial (NCT00322699) (including 22 patients) was treated with whole bladder laser light exposure as a replacement for radical cystectomy and Photofrin. In the treatment of superficial bladder cancer (non-muscle invasive), for patients who failed or were not suitable for traditional intravesical therapy, this protocol was used to assess the effectiveness and toxicity of three sequential whole PDT bladders with Photofrin and red laser lights (630 nm). Dose-limiting toxicity and disease progression were the outcomes that the authors aimed to measure. In 2016, Filonenko et al. [59] published the results of a multicenter prospective trial for the efficacy of a combination of transurethral resection (TUR) + PDT with alasens, which was given to the patients as an intravesicular instillation. The trial consisted of 45 individuals diagnosed with non-muscle invasive bladder cancer. A good treatment response was found, and no complications were observed. Of the 45 study patients, 35 (78%) completed a treatment follow-up of 12 months without relapse, and 22% experienced a recurrence. The results indicate that TUR with 5-aminolevulinic acid intraoperative PDT can provide an option for the treatment of non-muscle-invasive intermediate- to high-risk bladder cancer [59].

### 3.4. Gastroenterological Cancer

PDT is a highly promising tool for managing different solid malignancies, including gastrointestinal (GI) cancer. Surgery for GI cancer is connected with high morbidity and mortality, usually in elderly patients. PDT is unlikely to treat a large proportion of patients and is recommended for palliative care. Others may have early cancer but are unable to undergo surgery. Chemotherapy and additional radiation treatment are the only appropriate options for the majority of patients. PDT provides some benefits in terms of its greater safety and lower toxicity when treating malignant lesions over traditional GI cancer treatments. However, due to its low cost-effectiveness and the anatomical characteristics of the GI system, PDT is not commonly used to control upper GI cancer. Nevertheless, in early upper GI cancer patients who are subjected to high risk of curative surgical resection or systemic chemotherapy, PDT can be an effective alternative therapy. PDT has shown positive results in specific clinical trials with various upper GI cancers to boost treatment efficacy for upper GI cancer [29,60]. Esophageal cancer was among the first PDT treatment options approved in both the United States and Japan for endoscopic operations. In patients with an obstructive esophagus, PDT was first used as a local palliative treatment [61,62]. PDT is also suggested for the eradicative treatment of Barret’s esophagus, which is the prevalent condition of esophageal adenocarcinoma. In Japan, the use of PDT was authorized as a therapeutic option for superficial esophageal lesions with endoscopic resection [39]. PDT is also recommended for early local unresponsive cancers after radiotherapy by way of second-generation PS treatment, for which other therapies are difficult. Many GI tumors, including gastric cancer, biliary cancer, and pancreatic cancer, have also been investigated for PDT [63,64,65,66]. 

Several groups have published clinical trial results [64,67,68,69] for using the PDT technique to treat GI cancer, as shown in Table 2. In interventional study (NCT00060268) phase I and phase II trials, 11 patients with obstructive esophageal tumors were treated with 2-(1-Hydroxyethyl)-2-Devinylpyropheophorbide-a (HPPH). The purpose of this study was to assess the effectiveness of PDT with HPPH in treating patients who have obstructive esophageal tumors. Another group in an interventional study (a phase I trial including 18 patients with skin cancer), esophageal cancer was treated with PDT. The objective was to evaluate the safety and maximum tolerated dose of Photocyanine injection in the PDT of malignant tumors (especially skin cancer and esophageal cancer). In another interventional phase I trial (NCT00028405), 48 patients with liver metastasis, head and neck cancer, pelvic cancer, rectal cancer, sarcoma, colorectal cancer, breast cancer, or mouth cancer were treated with LS 11 (Taporfin Sodium) and a Lumaflex light delivery catheter. The objective was to evaluate the safety and tolerability of the intratumoral delivery of the oncolux system using non-coherent light for the photoactivation of LS 11 in refractory solid tumor patients.

## 4. Photoimmunotherapy for Solid Cancer 

**Figure 2:**
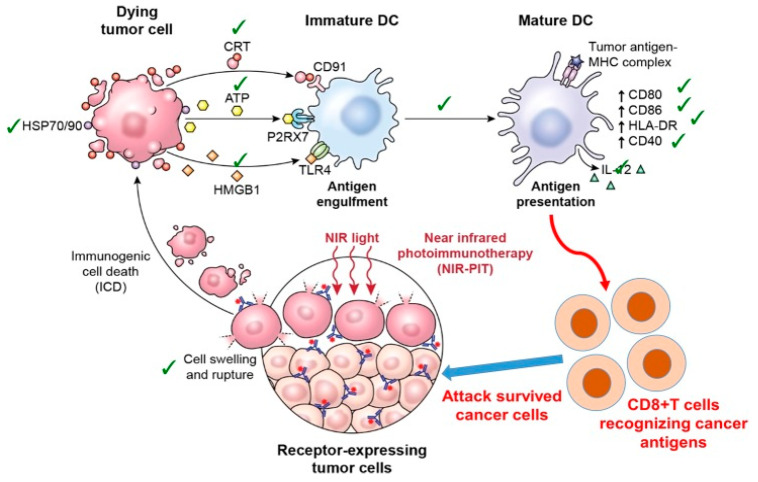
Near-infrared-Photoimmunotherapy (NIR-PIT) induce immunogenic cell death (ICD) biologically which enhances the antitumor host immunity for treating cancerous cells. Reproduced from [70].

**Figure 3:**
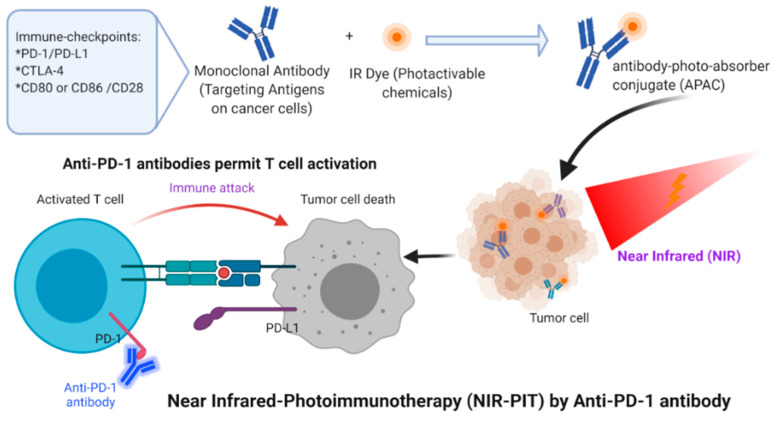
NIR-PIT for treating cancer cells. This therapy can involve injecting a monoclonal antibody conjugated with a photoabsorber followed by exposure to near-infrared light at the tumor site.

For half a century, three primary cancer therapies, surgery, radiation, and chemotherapy, have been the standard cornerstones of oncology care [70]. Cancer immunotherapy is a new approach, directed at T-cell stimulating cytokines, inhibitors of the immune checkpoints, depleting regulatory T (Tregs) cells, and cell-based treatments to control tumor growth selectively. Despite significant side effects, these approaches have proven beneficial in some cases. Nonetheless, modern immunotherapy for cancer does not lead directly to cancer cell death; rather, it kills cancer cells by cytotoxic immune cell activation [71,72]. Significant challenges of cancer will overpower the capacity of the human immune system to fight cancer. In the meantime, non-specific off-target immune system activation will cause auto-immune damage to healthy tissues. In principle, a treatment that kills cancer cells exclusively while triggering the localized host immune response would be perfect. One of several significant methods are used for enhancing the cytotoxicity of tumor-infiltrating T-cells through the inhibition of immuno-checkpoints, including the PD-1/PD-L1 axis or CTLA-4 [70]. When combining photothermal therapy with immunotherapy, the effects of phototherapy can provide a systemic treatment modality [73]. In addition, phototherapy has become a promising modality because it offers effective dose and light delivery for treating malignancies with PDT effects [73]. Combining both near-infrared (NIR) and immunotherapy, called photoimmunotherapy or (NIR-PIT), is a new molecularly targeted phototherapy for cancer that aims to selectivity stimulate the host’s immune response to kill cancer cells [74]. Essentially, unlike other conventional treatments, host defense against cancer is not be compromised but is also triggered by NIR-PIT-induced specific cancer cell death. Nonetheless, NIR-PIT’s highly immunogenic nature makes cellular death very rapid. This procedure is called immunogenic cell death (ICD), and NIR-PIT may be the best example of host immunity induction, as shown in Figure 2 and Figure 3.

NIR-PIT is based on injecting a near-infrared conjugate (IRdye700DX/IR700), a photoactivating chemical, and a monoclonal antibody (mAb), which is the target for expressed antigen on the cancer cell surface. The advantage of this technique over current therapies is that local NIR light exposure provides the rapid and highly selective ICD of targeted cancer cells, which can occur as early as 1 min after exposure to NIR light. This results in irreversible physical changes to the conjugated antibody/antigen complex, which is the reason for functional damage of the cell membrane [74]. Since the APAC primarily binds with cancer cells that over-express the desired cancer-associated antigen, light activation leads to the selective killing of cancer cells without destroying the surrounding normal cells. The mixture of the APAC and tumor light sensitivity is very precise and affects normal tissue to a minimal degree.

**Figure 4:**
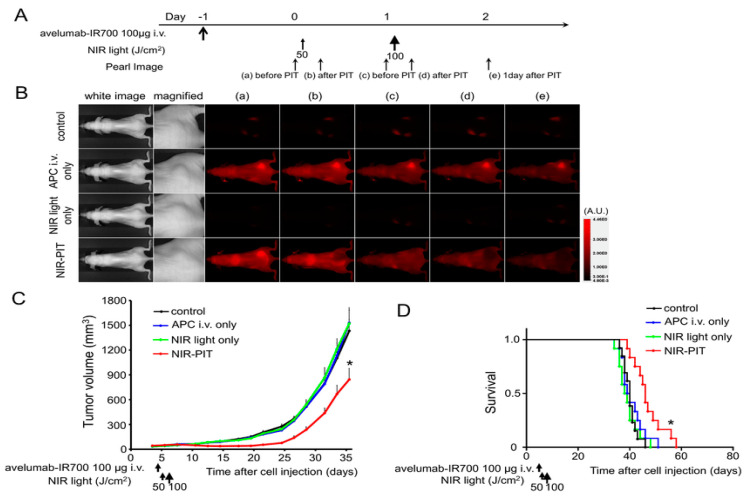
In vivo effect of NIR-PIT for treating animals bearing H441 lung cancer cells. (**A**) NIR-PIT injection regimen. (**B**) In vivo fluorescence real-time imaging of tumor-bearing mice in response to NIR-PIT. The tumors treated by NIR-PIT represented a decrease in IR700 fluorescence after NIR-PIT. (**C**) Tumor growth was substantially reduced in the NIR-PIT treatment groups. (**D**) Significantly prolonged survival was observed in the NIR-PIT treatment group. Reproduced from [75].

Preclinical research has shown that the NIR-PIT targeting of regulatory T-cells, a type of immunosuppressor cell within the tumor, to enhance tumor-cell-selective systemic host-immunity leads to significant responses in distant metastatic tumors that are not treated with light. Cancer-targeting NIR-PIT combined with cancer immunotherapies inhibits local tumors, metastasis, and may also inhibit recurrences, as shown in Figure 3. The first-in-human phase I/II clinical trial of NIR-PIT targeting EGFR using cetuximab-IR700 (RM1929) in patients with inoperable head and neck squamous cell cancer was successfully concluded in late 2017. A “fast-tracked” global phase III clinical trial began in 2019 (NCT03769506). Head and neck squamous cell carcinomas (HNSCC) are a type of immunosuppressive disease [73]. Cancer immunotherapy has been an excellent approach for treating solid tumors and can enhance the host antitumor response to HNSCC [73]. Earlier findings suggest that NIR-PIT is better than existing second- and third-line therapies for persistent cancers of the head and neck. In a preclinical study, Nagaya et al. [75] investigated the efficacy of NIR-PIT using avelumab (anti-PD-L1 monoclonal antibody) conjugated to the photo-absorber (IR700DX) on the H441 lung adenocarcinoma cell line. After NIR application, specific bindings were demonstrated and cell-specific deaths were observed. Avelumab-IR700 demonstrated a high concentration at the tumor site and high tumor/background ratios in the in vivo study. Four groups of animals bearing lung cancer were tested, as shown in Figure 4: (1) without treatment; (2) 100 μg of avelumab-IR700 i.v.; (3) NIR light exposure only; (4) 100 μg of avelumab-IR700 i.v. plus NIR light. In comparison with the other groups, the tumor growth was significantly reduced with NIR-PIT and significantly extended overall survival. The authors suggested that Avelumab-IR700 NIR-PIT is a suitable choice to treat tumors that can quickly be applied to individuals utilizing PD-L1-expression. 

## 5. Nanotechnology in PDT

Because of the extraordinary growth of research and applications in the nanotechnology field in recent decades, nanoparticles are suggested to enhance the diagnosis and treatment of various cancer types [76]. Today, some nanosized compounds, especially for cancer therapy, are being investigated for drug delivery [77]. For cancer drug delivery research and development, both in vivo and in vitro, nanomaterials with tailored properties can, therefore, be valuable to interact effectively with cellular components or mimic different chemical and biological characteristics [78]. Nanobiotechnology may help to develop diagnostic tools, contrasts, and drug delivery agents, as well as many other products [79]. The development of carrier systems to improve the solubility of medicinal products, shield them from degradation, and ensure improved delivery directly into tumor microenvironments is a promising approach to overcome the obstacles in drug delivery [80]. In this context, nanocarriers need to provide sufficient therapeutic efficacy, and issues such as unfavorable biodistribution or rapid drug clearance from tumor areas must be resolved [81]. Furthermore, nanocarriers will experience a prolonged presence in the blood flow, aggregate in the tumor microenvironment, and facilitate efficient drug uptake by cancer cells [82]. 

### 5.1. Advantages of Nanocarriers for PDT

**Figure 5:**
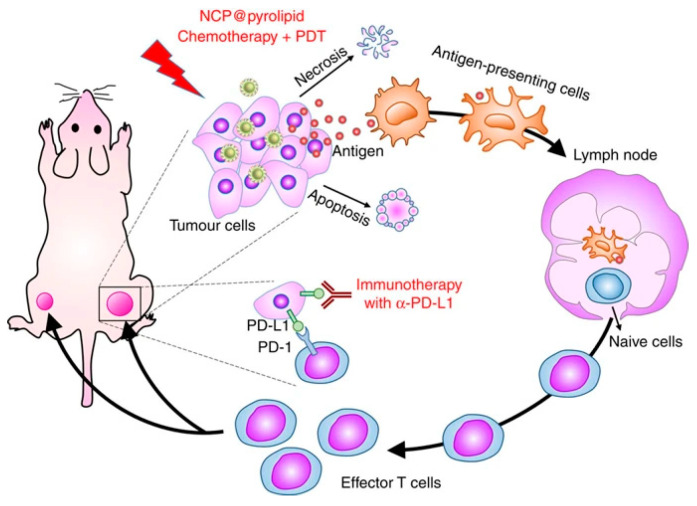
Chemotherapeutic agent and PDT of NCP@pyrolipid induced ICD, which caused the release of tumor-associated antigens (TAAs). Combined with the PD-L1 antibody inhibitor, the NCP@pyrolipid chemotherapy/PDT significantly stimulated the generation of tumor-specific effector T cells and improved their infiltration in both primary and distant tumors, resulting in tumor removal in the primary sites and distant tumors. Reproduced from [87].

While modern PDT has substantially improved the quality of life and increased the overall survival of cancer patients, more improvements in the therapeutic effectiveness of patients is vital to eliminate some of the most notable side effects (e.g., hydrophobic PSs and off-target side effect) [83,84]. Researchers have recently been exploring new strategies to improve the performance of PDT PSs, such as the potential to supplement PDT PSs with nanotechnology to enhance their efficiency [12]. The utilization of nanoparticles to maximize the effectiveness of PDT is encouraging because (1) it is possible to modify the huge surface areas of such nanoparticles with functional groups for targeting overexpressed specific proteins on cancer cells, as shown in Figure 5; (2) the volume of their distribution is high, and cells usually uptake drugs efficiently; (3) regulating drug release is feasible; (4) several biocompatible strategies enable hydrophobic drugs to be transferred through the blood; and (5) preferential accumulation is possible in the solid tumor area due to an enhanced permeability and retention (EPR) effect. PSs are either inserted into nanocarrier structures via covalent or noncovalent interactions in the nanoparticle (NP)-based drug delivery carriers or can be conjugated [85]. The high surface to volume ratios and high capacity for filling NPs with PSs are significant benefits of incorporating PS drugs into NPs [86]. Nanotechnology is desirable in the field of PDT for three main reasons: (1) the ability of the PS concentration in the desired area to be increased and the toxic effects on healthy tissue to be decreased; (2) the solubility of hydrophobic PS can be improved via nanoparticles; (3) due to zero-order release kinetics of some PS, NPs will maintain a consistent rate of PS distribution at the targeted sites [85].

### 5.2. Innovative Strategies of Using Nanocarriers in PDT 

**Figure 6:**
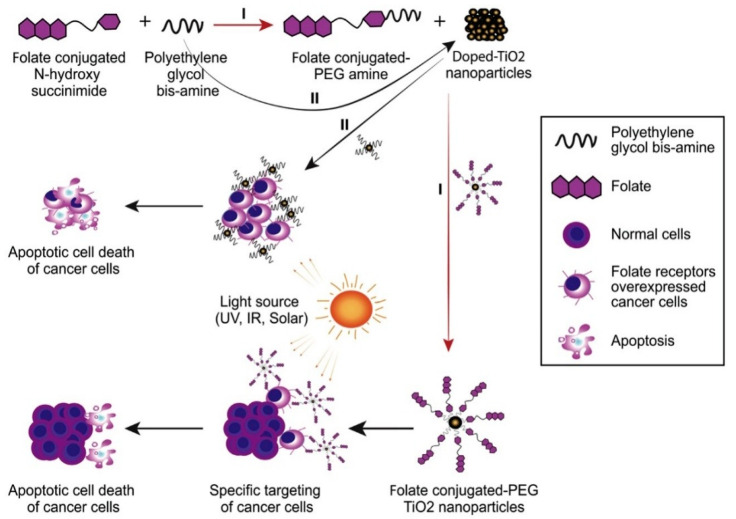
PEGylated doped- and undoped-TiO_2_ nanoparticles for cancer-related PDT. Mode of the conjugation of NPs against the cytotoxicity on cervical cancer cells. Reproduced from [91].

Several nanoparticles, including inorganic oxides, metal NPs, and porous and biodegradable polymer nanomaterials (Table 3) have been developed with potential applications for PDT to meet the requirements for the optimum delivery of PSs [28]. Drug delivery systems have been made using various degradable polymers, including polysaccharides and proteins [88]. Alginate, chitosan, dextran, albumin, gelatin, collagen, and agars are widely used as natural biodegradable polymers. Synthetic polymers such as aliphatic polylactide (PLA), polyglycolide (PGA), and their copolymer poly (D,L-lactide-co-glycolide) (PLGA) have been used as nondegradable multifunctional nanoplatforms for drug delivery. Polyacrylamide polymers, metallic nanoparticles, gold nanoparticles, PEGylated gold nanoparticles, and magnetic nanoparticles such as iron oxide have also been utilized as an example shown in Figure 6. The following are passive biodegradable organic NPs: liposomes, dispersions of oil, dendrimers, and polymeric NPs; ceramic-based NPs, such as Silica, Alumina, and Titania are considered passive, non-biodegradable organic NPs [89,90].

Nonetheless, recent studies have shown that PS drugs with the passive absorption of NPs can cause acute hypersensitivity in healthy cells since they cannot distinguish only cancerous cells. Therefore, PSs sometimes spread to healthy tissues [107,108]. Thus, in recent years, a major effort has been made to synthesize and classify the cancer cell targets of PS-NP bioconjugates that are only selectively incorporated into certain cancer cells to increase PS-NP therapeutic outcomes with specificity and eliminate undesirable phototoxic passive absorption effects [109]. Inorganic NPs may also be functionalized with ligands or antibodies to further improve PDT’s targeting activity against tumors by removing any PS cytotoxic effects on healthy cells [86,109]. In addition, inorganic NP quantum dots and metal-based NPs have unique physical properties that can be tailored to improve their capacity to activate the PS medications that they carry and also activate PS light within the optimal therapeutic wavelength of PDT; they can also be converted to release high-energy photons upon excitation by low infrared energy [108,110,111]. 

**Figure 7:**
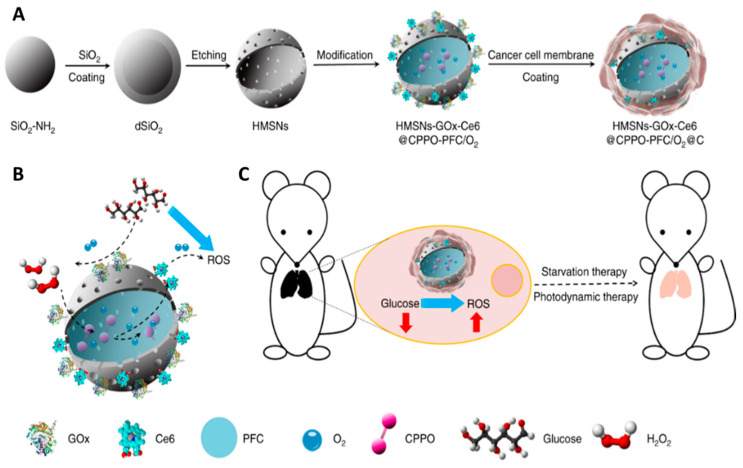
Schematic illustrations of the process for synthesizing a biomimetic nanoreactor: (**A**) ROS generation based on chemiluminescence resonance energy transfer (CRET) with glucose consumption, no light excitation (**B**), and synergetic photodynamic-starvation therapy for metastases (**C**). Reproduced from [115].

Biodegradable nanocomposites are polymers that are mostly hydrolyzed enzymatically in a biological environment, thus releasing photosensitive substances, as shown in Figure 7. The use of non-biodegradable nanoparticles prevents the release of PSs from nanoparticles, and the free diffusion of oxygen in and out of nanoparticles is necessary [112]. Due to their advantages in controlling the release of drugs, their flexibility in material production processes, and their high drug loading, biodegradable polymer-based nanoparticles are highly attractive. To achieve the necessary biocompatibility, degradation rate, and drug release patterns, their surface characteristics, morphology, and composition are optimized. The role of non-biodegradable nanoparticles in PDT is different because these nanoparticles are unable to controllably degrade and release drugs. They are not damaged by the treatment process and can, therefore, be used with appropriate stimulation frequently.

To boost PDT, functionalized NP systems are also used to facilitate the delivery of PS drugs into cancer cells by passive diffusion or active targeting [90]. The possibility to conjugate nanoparticles with targeting functional groups such as peptides, proteins, carbohydrates, antibodies, or folic acid may further improve the precise transportation of nanomedicines to their desired tumor sites. All these factors make nanomaterial delivery systems an attractive alternative to conventional PDTs, as such systems provide customizable PS transport, localization, and photodynamic reaction duration [81,113]. Most clinically licensed PSs also suffer from poor bioavailability and undesirable biodistribution. The increased EPR effect of dysfunctional tumor neovascular structures enables both the distribution and retention of PS nanocarriers at the cancer site. Nanoparticles can act as payload vehicles by using the EPR effect to further enhance the cellular uptake, transport, biodistribution, bioavailability, and pharmacokinetics of photoactive agents [114]. For the nanoparticles used in PDT, the primary objective is to increase effectiveness and reduce the amount of phototoxicity produced. For instance, He, C. et al. [87] synthesized immunogenic polymeric nanoparticles (NCPs) loaded with an oxaliplatin chemodrug and PS pyropheophorbide-lipid conjugate (pyrolipid); the authors called this nanocomposite NCP@pyrolipid, as shown in Figure 5. This nanocomposite showed the ability to combine chemotherapy with PDT for a great response besides enhancing the tumor immune response. Moreover, once combined with an immunotherapeutic agent (PD-L1 inhibitor antibody), NPs mediate the strong inhibition of both light-irradiated primary tumors and non-irradiated distant tumors by activating the immune response. 

## 6. Photothermal Therapy (PTT)

The Photothermal therapy, which is an extension of PDT, is a promising therapeutic approach using laser light in order to produce thermal damage in the area of interest (e.g., the tumor) [116]. The PTT approach has been utilized to address the problem NPs non-specific biodistribution [117]. Magnetic nanoparticles which has dual functionalities of near-infrared (NIR) absorption and magnetism such as gold or iron oxide NPs are special because they are capable of MRI contrast agents and can be placed in the target tumors with external magnetic forces while allowing NIR irradiation to be converted into PTT energy [117]. PTT is essential, in contrast to PDT, which has an antitumor activity based on the creation of radical oxygen species, and therefore oxygen occurs mainly by increasing the temperature of the environment and PTT does not require oxygen to generate its cytotoxic effect on cancerous cells. An important example of PTT is the Laser interstitial thermal treatment (LITT) which is one of the PTT strategies that has been checked for a cytoreduction effect on brain malignancies [118]. In a recent, multicenter, cohort study compared findings of newly diagnosed glioblastoma (GBM) patients with LITT to a biopsy followed by conventional chemotherapy and radiation therapy. The authors also noted that almost the entire blue coverage of thermal damage thresholds or TDT-lined patients with LITT had better survival relative to those with biopsy alone or in combination. LITT may also provide a desirable option, as an invasive cytoreductive technique, to biopsy alone in patients that have trouble reaching tumors or cannot handle craniotomy [119]. The recent interest in LITT has led to many reports describing neurosurgical pathology efforts, including neoplasms and epileptogenic foci [120,121]. LITT has now been well known in treating certain pathologies including chronic GBM and epileptogenic foci. Data on LITT emerging applications are very promising and procedural indicators continue to develop [118]. 

## 7. Conclusions and Future Directions

The field of PDT has evolved rapidly and is continuously being evaluated with new techniques. To make PDT more active and selective, molecular strategies are being developed. For the targeted and effective delivery of photosynthetic medicines, many organic and inorganic NPs have been produced. This review shows that NPs can provide solutions to address the critical limitations of the delivery of traditional PS medications, thereby improving the overall effectiveness of the treatment of PDT cancer. The water solubility of hydrophobic PS drugs can be improved with nanostructures mixed with PS drugs. Further, NPs can enhance the targeting ability of PS drugs using the EPR effect to target cancer cells selectively. The preferential deposition of PS drugs in tumors further increases by functionalizing the surfaces of NPs and conjugating them with targeting ligands. Despite the considerable developments involved in making adjusted nanomaterials for efficient PDT cancer treatment, progress in developing a nanosized pharmaceutical delivery system based on surface-functionalized NPs integrating directed tumor molecular interactions with the successful generation of ROS from PSs irradiated by PDT remains challenging [122,123].

To improve PDT’s efficacy for cancers, nanoparticles with active and passive functional roles have been developed. In the last few years, numerous molecules with various architectures, forms, and nanoscale sizes have become available to scientists due to the large developments in nanotechnology and nanoscience. All these factors contribute to the use of nanostructured compounds as a delivery system for PSs in PDT. Several studies show that nanoparticles can boost PS solubility, protect against degradation, modulate biodistribution, and lengthen blood half-life. Such properties offer promise for the introduction of advanced anticancer PDT, which offers an exciting alternative to the most commonly used methods. However, the stage of in vivo tests has only been reached by a small number of nanocarriers reported for PDT. This is a major limitation in the path to clinical application. Nanosystems that have provided positive results in cell line models during in vitro studies may be unstable or ineffective, with significantly altered characteristics and decreased phototoxic activity. Much more research must be conducted on the PDT—nanoparticle interface. The real clinical influence of these advanced nanosystems is anticipated in the next decade, as the integration of PDT with nanotechnology for medical applications may have a promising future for cancer treatment.

## Figures and Tables

**Table 1:** Clinical trials of the photodynamic therapy of brain tumors, lung cancers, and bladder cancers.

#	Clinical Trial Phase (NCT)	Status	Type of Cancer or Indication	Drug Used/Light Applicator	Sponsor
**1**	Phase I (NCT01682746)	completed	Brain tumor (Recurrent)	Photofrin (porfimer sodium) & PDT	Harry T Whelan, MD Pinnacle Biologics, Inc.
**2**	Not provided (NCT02632084)	completed	Pituitary Neoplasms	Not Provided	The Leeds Teaching Hospitals NHS Trust
**3**	Phase III (NCT00118222)	completed	Brain and CNS Tumors	Drug: porfimer sodium + adjuvant therapy + conventional surgery	Case Comprehensive Cancer Center National Cancer Institute (NCI)
**4**	Phase I (NCT00002647)	** Unknown	Brain and CNS Tumors Metastatic Cancer	Drug: verteporfin Procedure: conventional surgery	Medical College of Wisconsin
**5**	Phase III (NCT00003788)	** Unknown	Brain and CNS Tumors	Drug: carmustine, lomustine porfimer sodium, procarbazine hydrochloride Procedure: neoadjuvant therapy, surgical procedure. Radiation: radiatiotherapy	Colorado Health Foundation
**6**	Not provided (NCT00984243)	Completed	Lung cancer	Photofrin II of intravenously. An argon-dye or an excimer-dye laser (620–630 nm)	Mayo Clinic
**7**	Phase II (NCT00601848)	Active, not recruiting	Lung Cancer and Metastatic Cancer	Chemotherapy porfimer sodium	Abramson Cancer Center of the University of Pennsylvania
**8**	Not provided (NCT00754910)	Unknown	Lung cancer	porfimer sodium	Ohio State University Comprehensive Cancer Center
**9**	Phase I (NCT00025571)	Completed	Lung cancer	HPPH	Roswell Park Cancer Institute
**10**	Phase I (NCT01854684)	Recruiting	Recurrent NSCLC Stage IIA, IIB, IIIA, and IIIB NSCLC	Temoporfin	Roswell Park Cancer Institute
**11**	Phase I (NCT00526461)	Completed	NSCLC in Situ or NSCLC Microinvasive Bronchogenic Carcinoma	HPPH	Roswell Park Cancer Institute
**12**	Phase I (NCT01668823)	Completed	Adenocarcinoma of the Lung; Large Cell Lung Cancer; Recurrent NSCLC; Squamous Cell Lung Cancer; Stage 0 NSCLC	HPPH + PDT	Roswell Park Cancer Institute
**13**	Phase I (NCT00014066)	Completed	Lung cancer	Drug: hematoporphyrin derivative Radiation: brachytherapy	Roswell Park Cancer Institute
**14**	Phase I (NCT02916745)	Not yet recruiting	NSCLC	Porfimer sodium	Concordia Laboratories Inc.
**15**	Not provided (NCT01842555)	Recruiting	Lung Cancer; Esophageal	PDT	Main Line Health
**16**	Phase II (NCT00054002)	Completed	Malignant Mesothelioma	Procedure: adjuvant therapy Procedure: conventional surgery Drug: porfimer sodium	Roswell Park Cancer Institute
**17**	Phase I (NCT02464761)	Recruiting	Vertebral Metastases	Visudyne	Sunnybrook Health Sciences Centre
**18**	Not provided (NCT02514226)	Not yet recruiting	Bronchiectasis; Periodontal Disease	PDT	University of Nove de Julho
**19**	Phase II (NCT02497053)	Recruiting	Malignant Pleural Mesothelioma	Pemetrexed/platinum Chemotherapy	Ain Shams University
**20**	Phase I (NCT03053635)	completed	Non-Muscle Invasive Bladder Cancer (NMIBC) Refractory to BCG	Drug: TLD1433 infusion and photodynamic therapy (PDT) treatment	Theralase Inc. University Health Network, Toronto Medelis Inc. WCCT Global
**21**	phase II	Recruiting	Non-Muscle Invasive Bladder Cancer (NMIBC) Refractory to BCG	TLD-1433 Bladder infusion + PDT	Theralase Inc.
**22**	Phase I (NCT01303991)	Active, not recruiting	Intermediate or High-risk Bladder Cancer	Hexvix PDT with Karl Storz T-Light	Photocure Karl Storz
**23**	phase I phase II (NCT00322699)	completed	Superficial Bladder Cancer	Procedure: Whole bladder laser light treatment as an alternative to radical cystectomy. Drug: Photofrin	North Florida/South Georgia Veterans Health System North Florida Foundation for Research and Education Axcan Pharma

Note: Data were gathered by searching the National Institutes of Health (NIH)’s Clinical Trials.gov database at https://clinicaltrials.gov/. This Table includes information on clinical trials as of 19 May 2020. ** Unknown: The study has passed its completion date, and status has not been verified in more than two years.

**Table 2:** Clinical trials of photodynamic therapy of head and neck cancer and gastrointestinal tract cancer.

#	Clinical Trial Phase (NCT#)	Status	Type of Cancer or Indication	Drug Used	Sponsor
**1**	Phase I, II (NCT02070432)	Recruiting	Head and neck cancer	LUZ11	Luzitin SA
**2**	Phase I (NCT00978081)	Active, not recruiting	Head and Neck Cancer Precancerous Condition	aminolevulinic acid hydrochloride	Abramson Cancer Center of the University of Pennsylvania
**3**	Phase II (NCT00003856)	** Unknown	Head and Neck Cancer	temoporfin	Quintiles, Inc.
**4**	Phase I (NCT01019954)	Completed	Head and Neck Tumors	Levulan	Abramson Cancer Center of the University of Pennsylvania
**5**	Phase I (NCT00670397)	Completed	Head and Neck Cancer Precancerous/Nonmalignant Condition	porfimer sodium + PDT	Roswell Park Cancer Institute
**6**	Phase I (NCT00028405)	Completed	Liver Metastasis Pelvic Cancer Head and Neck Breast, Colorectal, rectal and Mouth cancer, sarcoma.	Drug: LS 11(Taporfin Sodium) Device: Lumaflex Light Delivery Catheter	Light Sciences LLC
**7**	Phase I (NCT01043016)	** Unknown	Skin Cancer Esophageal Cancer	Photocyanine Injection	Fujian Longhua Pharmaceutical Co. Ltd
**8**	Phase II (NCT01086488)	** Unknown	Nasopharyngeal Carcinoma	FOSCAN	Ministry of Health, Malaysia
**9**	Phase I, II (NCT00060268)	Completed	Esophageal Cancer	HPPH	Roswell Park Cancer Institute
**10**	Phase II (NCT00002935)	Completed	Esophageal Cancer	porfimer sodium	Roswell Park Cancer Institute
**11**	Phase III (NCT02628665)	Recruiting	Stage I, II and III of Esophageal Adenocarcinoma and Esophageal Squamous Cell Carcinoma	photosensitizer(photofrin) Device: 630 nm laser irradiation (DIOMED)	The First Affiliated Hospital of Henan University of Science and Technology
**12**	Phase II (NCT00217087)	Completed	Early Stage Esophageal Adenocarcinoma. Barrett Esophagus	Porfimer sodium 2 mg/kg	Mayo Clinic
**13**	Phase I (NCT01366833)	** Unknown	Malignant Dysphagia; Esophageal Cancer	Radiation: Brachytherapy; Procedure: Stent insertion	McGill University Health Center
**14**	(NCT00587314)	Enrolling by invitation	Barrett’s Esophagus; Early Esophageal Adenocarcinoma	Biopsy	Mayo Clinic
**15**	Phase IV (NCT00155337)	Completed	Oral Leukoplakia	Not provided	National Taiwan University Hospital

Note: Data were gathered by searching the National Institutes of Health (NIH)’s Clinical Trials.gov database at https://clinicaltrials.gov/. This Table includes information on clinical trials as of 19 May 2020. ** Unknown: The study has passed its completion date, and its status has not been verified in more than two years.

**Table 3:** Types of nanoparticles used to enhance the efficacy of photodynamic therapy.

Type of Nanoparticles	Drug Used	Targeted Cancer	Results	Reference
doped- and undoped-TiO_2_ NPs stabilized by PEG	Titanium dioxide	cervical cancer cells (HeLa)	NPs significantly reduced the survival of human cervical cancer cells (HeLa).	[91]
nanoscale metal-organic frameworks (UiO-66-H/N3 NMOFs)	azido-/photosensitizer-terminated UiO-66 + the bioreductive prodrug banoxantrone (AQ4N)	not specified	Enhanced therapeutic efficacy Reduced systemic toxicity	[92]
gold nanoparticle (AuNP) conjugated photodynamic therapy (PDT) in combination with cannabidiol (CBD)	Cannabidiol (CBD)	breast cancer	Reduced side effects and toxicity to normal cells PDT and CBD are promising for hindering breast cancer progression and development	[93]
conjugation of gold nanoparticles (GNPs)	5-aminolevulinic acid (5-ALA)	cutaneous squamous cell carcinoma (cSCC) non-melanoma skin cancer	PDT with 5-ALA and GNPs-conjugated 5-ALA (5-ALA-GNPs) significantly suppressed cell viability and increased cell apoptosis and singlet oxygen generation in both HaCat and A431 cells	[94]
metal-based nanoparticles (NPs)	NiO NPs	cervical cancer cells (HeLa)	A light dose of 100 J/cm^2^ and a NiO NP concentration of 180 μg/mL exhibited an effective PDT outcome on cervical cancer cells. The photokilling effect of NiO NPs as a potential treatment for cervical malignancy was supported	[95]
(PCN-Fe(III)-PTX) nanoparticles (NPs)	Fe(III)-complexed	pancreatic cancer (PaC)	NPs represented an ideal agent for mediating effective MRI-guided chemotherapy-PDT; great promise for the clinical treatment of pancreatic cancer	[96]
peptide p 18-4/chlorin e6 (Ce6)-conjugated polyhedral oligomeric silsesquioxane (PPC) nanoparticles	Chlorin e6 (Ce6)	breast cancer cells	PPC NPs are highly effective PDT agents for breast cancer therapy.	[97]
Stem cell membrane –camouflaged bioinspired nanoparticles	Chlorin e6 (Ce6)-	lung cancer	Enhanced antitumor effect of Ng/Ce6@SCV after NIR irradiation by significantly suppressing primary tumor growth with fewer side effects.	[98]
Gefitinib PLGA nanoparticles (GNPs)	5-aminolevulinic acid (5-ALA)	lung cancer	The synergistic effect of CPDT was confirmed	[99]
graphene oxide nanoparticles	Polyethylene glycol (PEG), folic acid (FA), PS indocyanine green (ICG), and doxorubicin.	osteosarcoma	NPs with combined Chemo–PDT inhibited the proliferation and migration of osteosarcoma cells.	[100]
TID nanoparticles	Doxorubicin (DOX)	breast cancer	TID NPs rapidly destroyed the genetic substances and potently induced the apoptosis of breast cancer cells.	[101]
Fe_3_O_4_ nanoparticles (IONs)	Trastuzumab	breast cancer	No cytotoxicity was observed after incubating MCF 7 cells under various Fe concentrations of nanoparticles and Theranostic agents.	[102]
P123 Pluronic^®^-based nanoparticles	Hypericin	cervical cancer	HYP/P123 micelles had effective and selective time- and dose-dependent phototoxic effects on cervical cancer cells	[103]
photoactivatable Pt(IV) prodrug-backboned polymeric nanoparticle system (CNPPtCP/si(c-fos))	Platinum	ovarian Cancer	CNPPtCP/si(c-fos) displayed excellent synergistic therapeutic efficacy on PROC with low toxicity.	[104]
poly-ε-caprolactone nanoparticles (PCL NPs)	IR780 and paclitaxel (PTX)	ovarian cancer	LHRH peptide modified PCL (PCL-LHRH) NPs demonstrated increased internalization in ovarian tumor cells in vitro and selective targeting in tumor xenografts in vivo.	[105]
Hyaluronic acid (HA) coated polymeric nanoparticles (HA-NPs)	docetaxel (DTX) and PS meso-tetraphenyl chlorine disulfonate (TPCS2a)	breast cancer	Combination therapy using co-loaded NPs (HA@DTX/TPCS2a-NPs) had superior efficacy over monotherapies (HA@DTX-NPs or HA@TPCS2a-NPs) in reducing the self-renewal capacity and eradicating the CSC population evaluated with an aldehyde dehydrogenase activity assay and CD44/CD24 immunostaining.	[106]
